# Systematic analyses reveal uniqueness and origin of the CFEM domain in fungi

**DOI:** 10.1038/srep13032

**Published:** 2015-08-10

**Authors:** Zhen-Na Zhang, Qin-Yi Wu, Gui-Zhi Zhang, Yue-Yan Zhu, Robert W. Murphy, Zhen Liu, Cheng-Gang Zou

**Affiliations:** 1 Laboratory for Conservation and Utilization of Bio-Resources, Yunnan University, Kunming, China; 2 Xiamen Tobacco Industrial CO., LTD, Xiamen, China; 3 State Key Laboratory of Genetic Resources and Evolution, Kunming Institute of Zoology, Chinese Academy of Sciences, Kunming, China

## Abstract

CFEM domain commonly occurs in fungal extracellular membrane proteins. To provide insights for understanding putative functions of CFEM, we investigate the evolutionary dynamics of CFEM domains by systematic comparative genomic analyses among diverse animals, plants, and more than 100 fungal species, which are representative across the entire group of fungi. We here show that CFEM domain is unique to fungi. Experiments using tissue culture demonstrate that the CFEM-containing ESTs in some plants originate from endophytic fungi. We also find that CFEM domain does not occur in all fungi. Its single origin dates to the most recent common ancestors of Ascomycota and Basidiomycota, instead of multiple origins. Although the length and architecture of CFEM domains are relatively conserved, the domain-number varies significantly among different fungal species. In general, pathogenic fungi have a larger number of domains compared to other species. Domain-expansion across fungal genomes appears to be driven by domain duplication and gene duplication via recombination. These findings generate a clear evolutionary trajectory of CFEM domains and provide novel insights into the functional exchange of CFEM-containing proteins from cell-surface components to mediators in host-pathogen interactions.

Protein domains are fundamental components of protein structure and function, and also the smallest units of evolution^1^,^2^. The number of domains varies from one to more than 10 among different proteins^3^. In general, eukaryotic proteins tend to have more domains than those in prokaryotes^4^. The molecular mechanisms underlying gains and losses of domains have been widely documented. Primarily, these consist of retroposition, gene fusion through the joining of exons from adjacent genes, DNA recombination and duplication^5^,^6^. The number of domains within a protein varies considerably among genomes of different organisms and non-homologous proteins may contain the same domain. Thus, the evolutionary trajectories of protein domains can provide insights into the roles functional changes of domains play during the courses of evolution.

CFEM is a protein domain containing eight cysteines, which characteristically distinguish it from known cysteine-rich domains^7^. The consensus motif deduced for CFEM domain is as follows: PxC[A/G]x_2_Cx_8-12_Cx_1-3_[x/T]Dx_2-5_CxCx_9-14_Cx_3-4_Cx_15-16_, where x is any residue, and its range is indicated. CFEM was first identified in ACI1, an adenylate cyclase (MAC1)-interacting protein in *Magnaporthe grisea*, the causal agent of rice blast disease^7^. MAC1 is a key component for generating a specific structure of appressorium, which is essential for *M. grisea* to infect rice^8^. These results imply that the CFEM-containing protein ACI1 may also play an essential role in the infection of *M. grisea*. In addition, evidence points to some proteins containing CFEM also being involved in fungal pathogenesis, such as Pth11 in *M. grisea*^9^ and CSA1 in *Candida albicans*^10^. However, some proteins in non-pathogenic fungi also contain CFEM domains. For example, CCW14 in *Saccharomyces cerevisiae* contains a single CFEM domain, which is involved in cell wall biogenesis and plays an important role in maintaining the integrity and stability of the cell wall^11^,^12^. Thus, the genes encoding CFEM-containing proteins may be involved in different functional categories.

Although CFEM has been identified in a few genes for more than ten years, its genomic pattern and evolutionary trajectory remain largely unknown. To understand the evolution of CFEM-function, we compile and evaluate more than 100 genomic and proteomic sequences of fungi, animals, and plants from existing databases. We describe the genomic patterns of CFEM in these organisms and experimentally show that CFEM is unique to fungi. Our results further demonstrate that CFEM probably originated from the most recent common ancestors of Ascomycota and Basidiomycota. The pathogenic fungi contain larger numbers of CFEM than non-pathogenic ones. Finally, domain duplication and recombination appear to be the main genetic mechanisms of expanding CFEMs in fungal genomes.

## Results and Discussion

### CFEM domain unique to fungi

To characterize the CFEM domains, we searched the GenBank non-redundant database using 276 individual CFEM sequences from the Pfam database as queries. We performed reiterative PSI-BLAST searches due to the high variation of CFEM sequences, except for the consensus eight cysteine residues. We identified many proteins containing CFEM in various fungal species, but we failed to retrieve animal or prokaryotic sequences with CFEM. To further validate and expand these data, we performed TBLATN against the GenBank “est_others” database. A large number of fungal ESTs encoding CFEM-containing proteins were identified. Interestingly, we also found that three plant ESTs encoded proteins contain CFEM domains, one from corn and two from sorghum. These blast results were consistent with that from a previous study^7^.

However, when we blasted protein sequences translated by the three plant ESTs identified from maize and sorghum EST databases as queries against GenBank non-redundant database, the best hits were the protein sequences containing CFEM from *Fusarium oxysporum* (identity: 93%), *Nectria haematococca* (identity: 84%), and *Gibberella zeae* (identity: 61%). The approach failed to identify any sequences from the two plants. To further verify the results, we downloaded the genomes and cDNA sequences of maize and sorghum, and performed a local BLAST. No sequences obtained high identity to the queries. There are three possible explanations that may account for the results. First, the genes encoding the CFEM-containing proteins exist in the genomes of the two plants, but were not recovered during genome sequencing. This possibility is unlikely due to the low probability for coincidence of sequencing gap in two plants. Second, the genes containing CFEM domain in plants may be acquired from fungi species through horizontal gene transfer (HGT). In fact, it possibility is also low. Because when we employed the same queries that were used to succeed in identifying the ESTs containing CFEM in plant EST databases to blast the plant genomes, we failed to identify any hits with the conserved motif of CFEM and the respective genomic sequences. If the plants obtained CFEM by HGT, we should be able to identify these sequences because CFEMs have inserted into the plant genomes. Third, because these fungi are pathogens of maize, pea, and rice^13^,^14^,^15^, it is possible that the plant samples used to construct the EST databases were contaminated with these fungi.

**Figure 1:**
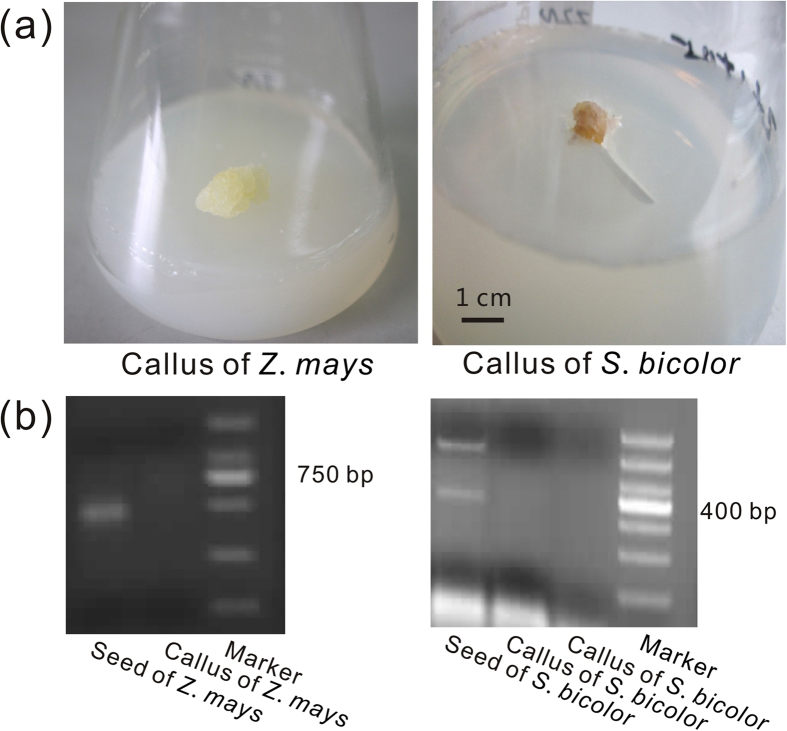
Amplification of DNA segments encoded CFEM domains in *Z. mays* and *S. bicolor*. (**A**) Callus tissues are induced from seeds of *Z. mays* and *S. bicolor*, respectively. (**B**) Target segments succeed in being amplified from the plant seeds, but not from the plant callus by PCR using the same primer pairs.

To verify this possibility, we conducted the plant tissue cultures for maize (*Z. mays*) and sorghum (*S. bicolor*) to exclude fungal contamination (Fig. 1A). We failed to amplify the sequences containing CFEM from genomic DNA of the calluses; in contrast we obtained the sequences containing CFEM from genomic DNA of non-treated plants using the same primers (Fig. 1B). These results indicated that the ESTs encoded CFEM-containing proteins in corn and sorghum originated from their endophytic fungi and further rejected the hypothesis that the CFEM genes in plants came from the fungi by HGT. Our results provided strong evidence for supporting that the CFEM domain is unique to fungi and redefined the distribution range of CFEM, which solved a previous puzzle why the domain widely distributed in fungi would occur in the plant EST databases^16^. More importantly, the results laid the foundation for understanding the evolutionary dynamics and putative function of CFEM in fungi.

### Origin of CFEM in fungi

To investigate the evolutionary trajectories of unique fungal CFEM domains, we downloaded up to 100 fungal genomes and corresponding proteomes were from public databases (Table S1). The selected genome sequences had high sequencing quality (>6 × coverage) and covered all fungal phyla. Using all 276 seed CFEM sequences from the Pfam database as queries, we searched all proteomes above by using the local reiterative PSI-BLAST. Searching identified 363 CFEM domains in 64 fungal species which belong to the phyla of Ascomycota and Basidiomycota. CFEM domains were absent in 36 species that represented the phyla of Zygomycota, Chytridiomycota and Microsporidia.

**Figure 2:**
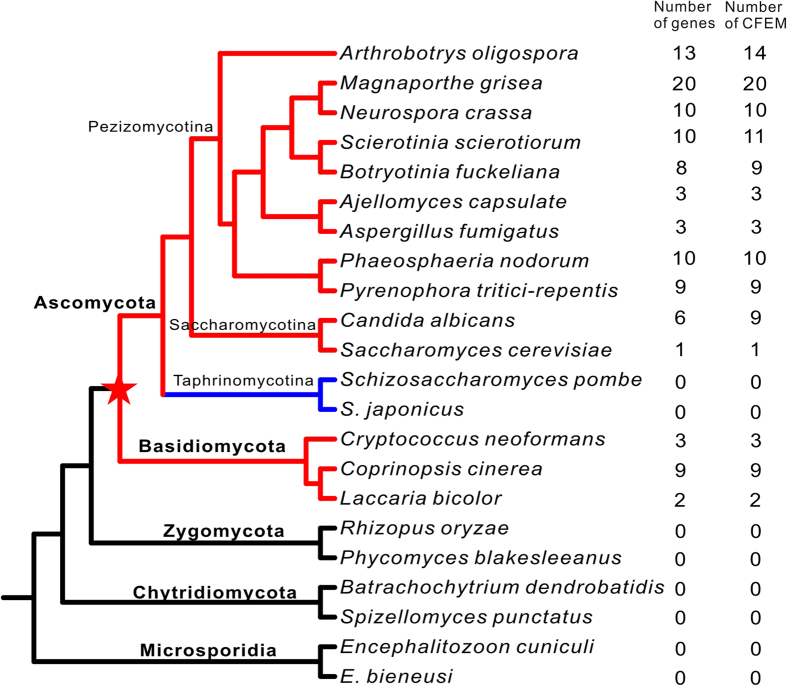
The number of genes containing CFEMs and how many CFEMs exist in each species mapped on the phylogeny of 22 selected fungal species. Red bWranches indicate the fungal species containing CFEMs and the blue branches indicate the fungal species that lost CFEMs in phylum Ascomycota.

**Figure 3:**
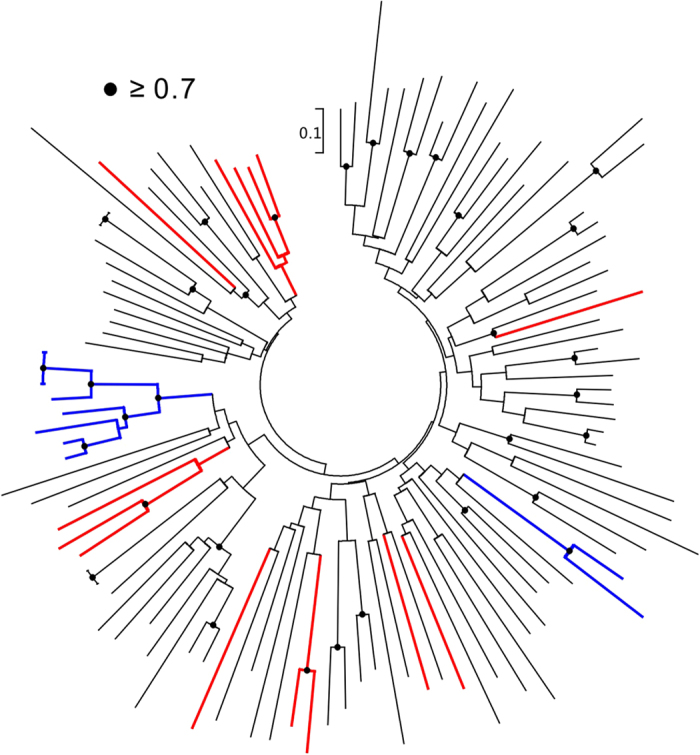
Neighbor-joining diagram constructed by using CFEM sequences from 22 selected fungal species. Red branches indicate species of Basidiomycota, blue branches species of Saccharomycotina, and black branches species of Pezizomycotina. Bootstrap values larger than 0.7 are shown in black circles.

To explore the origin of CFEMs in fungi, domains were mapped onto the phylogeny of fungi. To avoid unreliability due to ambiguous phylogenetic relationships among the 100 fungi, we selected 22 representative species (Fig. 2) which covered all fungal phyla with at least two species from each phylum, subphylum or class, and were the most divergent taxa within any group. Taxa expressing CFEM domains occurred in the Ascomycota and Basidiomycota (Fig. 2). The distribution of CFEMs displayed enormous diversity in the Ascomycota. No CFEM was identified in subphylum Taphrinomycotina and the number of CFEMs ranged from one in *S. cerevisiae* to 20 in *M. grisea*. Two competitive hypotheses explained the distribution pattern of CFEMs: (1) CFEM independently originated in the ancestors of Basidiomycota, Pezizomycotina and Saccharomycotina; or (2) they originated in the most recent common ancestor of Ascomycota and Basidiomycota and were lost in the ancestor of the Taphrinomycotina. To distinguish between these hypotheses, we reconstructed the phylogenetic relationships of 14 domains from Basidiomycota, 89 from Pezizomycotina, and 10 from Saccharomycotina. The CFEM domains scattered throughout the phylogeny instead of clustering together within the same phylum (Fig. 3). This indicated that the CFEMs evolved from a common ancestor, rather than having independent origins. If CFEMs occurred independently in the ancestors of Basidiomycota, Pezizomycotina and Saccharomycotina, descent domains would cluster together. Moreover, the former is more parsimonious in that independent gains require three steps vs. one loss. Consequently, CFEMs likely originated in the most recent common ancestor of Pezizomycotina, Basidiomycot, and Saccharomycotina and were lost in the ancestor of Taphrinomycotina. Our results for the first time revealed the origin position of CFEM and showed its evolutionary trajectory in fungi, providing insights into exploring the functional alteration of CFEM in fungal cell component and pathogenesis^16^. Nevertheless, the genetic and evolutionary mechanisms for CFEM origin in fungi are less clear. More genomic and experimental data are required for detecting this open question.

### Pathogenic fungi have significantly larger numbers of CFEMs

To further provide insights for the evolutionary characteristics of CFEMs, we compared all of the 363 CFEM domains identified in 64 fungal species and constructed the phylogenetic tree (Supplementary figure 1). Although the overall length of the CFEMs was quite conserved with ~60 amino acids, the number of CFEMs varied greatly among fungi. About 50% of the genomes in 23 species of Saccharomycotina contained one CFEM, only. In comparison, ~90% of the genomes in 33 species of Pezizomycotina contained ≥3 copies and ~70% of the genomes in 8 species of Basidiomycota contained ≥5 copies of CFEMs.

**Figure 4:**
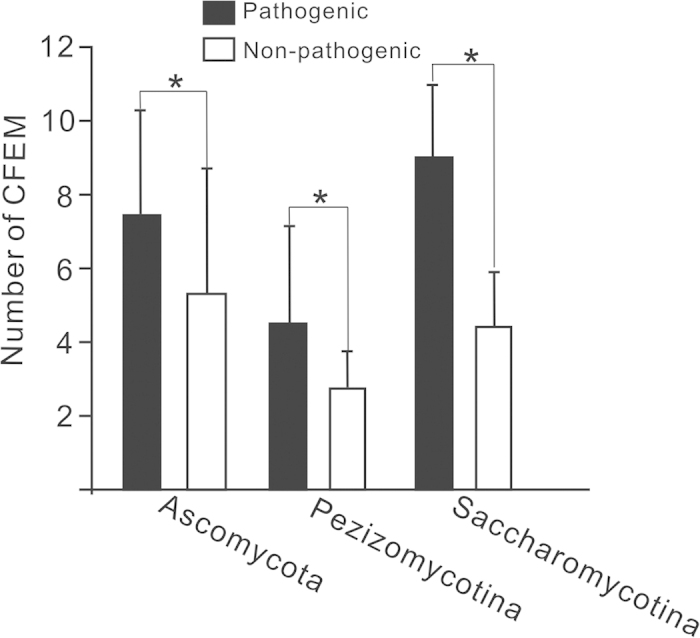
Numbers of CFEMs in pathogenic and non-pathogenic species in the phylum of Ascomycota, as well as the subphyla of Pezizomycotina and Saccharomycotina, respectively. Standard errors are shown by error bars. *P < 0.05.

The pathogenic species in fungi seemed to possess more CFEMs than non-pathogenic ones^16^. To determine the general pattern, we computed the numbers of CFEM in different phyla of fungi and examined their pathogenesis. The numbers of CFEMs of pathogenic species were significantly larger than those in non-pathogenic fungi in the phylum of Ascomycota, as well as the subphyla of Pezizomycotina and Saccharomycotina, respectively (*P* < 0.05, *t*-test; Fig. 4). The results supported the perspective that there was a positive correlation between CFEM domain occurrence and fungal pathogenicity. It is suggested that the increase in the number of CFEM may play important roles for fungal pathogenicity. In addition to the fundamental components for cell wall, genes containing CFEMs, particularly those containing multiple CFEM domains, appeared to participate in pathogenic mechanisms. For example, CCW14, the only CFEM-containing protein in *S. cerevisiae*, involves the formation of inner layer of the cell wall^11^,^12^. However, the CFEM-containing proteins RBT5, RBT51, and CSA2 are involved in the heme-iron utilization from human hemoglobin during *C. albicans* hyphal growth^17^,^18^. In addition, RBT51 and RBT5, together with another CFEM-containing CSA1, play a key role in the formation and maintenance of the biofilm structure in *C. albicans*^19^.

### Possible expansion mechanisms of CFEM

**Figure 5:**
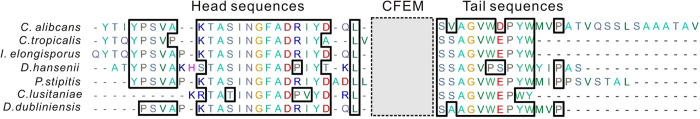
Conserved head and tail sequences of CFEM domains across different fungi.

To clarify the drivers of variation in the number of CFEMs across fungi, we explored the possible mechanisms of expansion. For genes containing multiple CFEM domains, domain duplication mediated by unequal crossing over is an important mechanism. For example, protein WAP1 in *C*. *albicans* contains four CFEM domains, called I, II, III, and IV according to the order along the gene sequence. The amino acid sequences of domains I and II are identical and only one replacement differentiates domains I and IV. Although the largest difference occurs between domain I and III, the identity of CFEM sequences is up to 83%, which is higher than that from other fungi. These domains most likely stem from a common ancestor. Further, the identity of the flanking sequences of the CFEM domains averages >90% (Fig. 5), which supports the scenario of unequal cross-over via mismatching between homologous chromosomes in cell division.

Gene duplication can also drive expansions of CFEMs. In this case, duplicated genes always tend to cluster together along the chromosomes. In *C. albicans*, genes 19.5635, 19.5636 and 19.5674 on chromosome 4 display tandem arrangement and all contain CFEMs. Their protein sequences are highly similar with the average identity of >85%. Thus, gene duplication and tandem duplication likely generate CFEM expansion in the genome. Notwithstanding, many other mechanisms can create new domains, such as are retroposition, gene fusion, and DNA recombination^5^,^6^. More evidence is required to infer their involvement in the expansion of fungal CFEMs.

## Conclusions

We demonstrate that CFEM domains are unique to fungi. Our analyses suggest that CFEM originated in the common ancestor of Ascomycota and Basidiomycota and was lost independently in Taphrinomycotina. The underlying reasons for the loss remain unclear. Our analyses reveal a significant association between the number of CFEM domains and fungal pathogenicity. The original function of CFEM domains is cell wall/membrane constitutions, but divergence facilitates various functions, such as pathogenicity. Finally, tandem duplication likely generates the possible expansion of CFEM domains.

## Materials and Methods

### Search and identification of CFEM

We extracted all of the putative CFEMs and obtained total 276 sequences (PF05730) from the Pfam database release 26.0^20^, which were identified as queries in further domain search. Although sequences exhibited greatdiversity all had the conserve motif containing eight cysteines: PxC[A/G]x_2_Cx_8-12_Cx_1–3_[x/T]Dx_2–5_CxCx_9–14_Cx_3–4_ Cx_15–16_C, where “x” indicated any residue^7^. Reiterative PSI-BLAST was performed to search with the queries against the GenBank non-redundant database to retrieve additional proteins containing CFEMs in other organisms. To investigate the CFEM profiles in fungi, we also downloaded a total of 100 fungal proteomes from public databases (Supplementary TableS1, Supplementary Material online), including BROAD-FGI (Fungal genome initiative, http://broadinstitute.org/science/projects/fungal-genome-initiative), JGI (DOE Joint Genome Institute, http://genome.jgi.doe.gov/genome-projects), and NCBI (http://blast.ncbi.nlm.nih.gov/). These fungal species covered all fungal phyla^21^. We conducted local reiterative PSI-BLASTs in these fungal genomes to identify the proteins containing CFEM. To further confirm the reliability of our searches, we submitted the PSI-BLAST results into Pfam search website (http://pfam.sanger.ac.uk/search) to highlight the CFEM in protein sequences with a E-vale cut-off of 10^−4^. We also performed TBLATN searches against the GenBank “est_others” database, which saved a large number of expressed sequence tags (ESTs), resulting in compensation and validation for the PSI-BLAT results.

### Phylogenetic analysis of CFEM domain

The deduced 113 amino-acid sequences of CFEM in 22 fungal species were initially aligned by MUSLE^22^ followed by manual adjustments. A tree depicting overall similarity of these sequences was reconstructed using the neighbor-joining method^23^ of MEGA6^24^ based on protein Poisson distances. Gaps in the alignment were not used in tree-building (complete-deletion option). The reliability of the nodes of the tree was evaluated by nonparametric bootstrapping^25^ using 1000 pseudo-replicates.

### Plant tissue culture

To investigate whether CFEM-containing proteins exist in plant^7^ or not, we cultured tissues for *Zea mays* and *Sorghum bicolor*. Seeds were surface-sterilized by immersion in 75% alcohol for 1 min and in 0.01% mercuric chloride for 5 min, and then kept in 4 °C for 48 h. These seeds were dissected by peeling off the seed coat and endosperm. Acquired cotyledons were cultured on Murashige and Skoog (MSO) medium at 25 °C in an incubator for about two weeks under darkness until calluses were generated. All operations were carried out under sterile conditions in a laminar flow hood. Non-sterilized seeds were used as controls and cultured in the same conditions.

We used RT-PCR to amplify CFEM from total RNA and genomic DNA isolated from the cultured tissues of *Z. mays* and *S. bicolor*. For the first-strand cDNA synthesis, 1 μg of total RNA was reverse transcribed in a volume of 20 μl and stored at −80 °C for further use. Two pairs of primers (5′-GCTATTCCTTGCCTTGACGA CGCC-3′, 5′-CCGAGACCCTTGAGGCCAGCAGC-3′ for *Z. mays*; 5′-GGACGCTGGCGGAGCCTGTG-3′, 5′-TTGCCGCTCAGGACTTTGGTGG-3′ for *S. bicolor*) were designed from CFEM sequences identified from ESTs of the two plants. All products were isolated from a 1.5% agarose gel and cloned using the T-vector. Positive clones were cycle sequenced in both directions using Big Dye Terminator (Applied Biosystems, Foster City, CA) on an ABI3730 sequencer.

## Additional Information

**How to cite this article**: Zhang, Z.-N. *et al.* Systematic analyses reveal uniqueness and origin of the CFEM domain in fungi. *Sci. Rep.* **5**, 13032; doi: 10.1038/srep13032 (2015).

## Supplementary Material

Supplementary Information
