# Supplementary material for: Systematic analyses reveal uniqueness and origin of the CFEM domain in fungi
Source: Sci Rep. 2015 Aug 10;5:13032. doi: 10.1038/srep13032 (PMC4530338; doi:10.1038/srep13032)
Supplement: Supplementary Information [file srep13032-s1.pdf]

Supporting Information

Systematic analyses reveal uniqueness and origin of the CFEM domain in fungi

Zhen-Na Zhang[1,2,]†, Qin-Yi Wu[1,]†, Gui-Zhi Zhang[2], Yue-Yan Zhu[1], Robert W. Murphy[3], Zhen Liu[3,]* and Cheng-Gang Zou[1,]*

[1]Laboratory for Conservation and Utilization of Bio-Resources, Yunnan University, Kunming, China

[2]Xiamen Tobacco Industrial CO., LTD, Xiamen, China

[3]State Key Laboratory of Genetic Resources and Evolution, Kunming Institute of Zoology, Chinese Academy of Sciences, Kunming, China

Table S1. Fungal species used in this study.

Species	Phylum	Genome source
Ajellomyces capsulata	Ascomycota	BROAD-FGI
Ajellomyces dermatitidis	Ascomycota	BROAD-FGI
Arthrobotrys oligospora	Ascomycota	NCBI
Arthroderma benhamiae	Ascomycota	BROAD-FGI
Arthroderma gypseum	Ascomycota	NCBI
Ascosphaera apis	Ascomycota	NCBI
Aspergillus carbonarius	Ascomycota	BROAD-FGI
Aspergillus clavatus	Ascomycota	BROAD-FGI
Aspergillus flavus	Ascomycota	BROAD-FGI
Asperigillus fumigates	Ascomycota	BROAD-FGI
Asperigillus nidulans	Ascomycota	BROAD-FGI
Asperigillus niger	Ascomycota	BROAD-FGI
Aspergillus oryzae	Ascomycota	BROAD-FGI
Aspergillus terreus	Ascomycota	BROAD-FGI
Botryotinia fuckeliana	Ascomycota	BROAD-FGI
Candida albicans	Ascomycota	BROAD-FGI
Candida dubliniensis	Ascomycota	NCBI
Candida glabrata	Ascomycota	NCBI
Candida parapsilosis	Ascomycota	BROAD-FGI
Candida tropicalis	Ascomycota	BROAD-FGI
Chaetomium globosum	Ascomycota	BROAD-FGI
Clavispora lusitaniae	Ascomycota	NCBI
Coccidioides immitis	Ascomycota	BROAD-FGI
Coccidioides posadasii	Ascomycota	BROAD-FGI
Cochliobolus heterostrophus	Ascomycota	JGI
Debaryomyces hansenii	Ascomycota	BROAD-FGI

Eremothecium gossypii	Ascomycota	NCBI
Fusarium oxysporum	Ascomycota	BROAD-FGI
Gibberella moniliformis	Ascomycota	NCBI
Gibberella zeae	Ascomycota	NCBI
Glomerella graminicola	Ascomycota	NCBI
Hypocrea jecorina	Ascomycota	NCBI
Hypocrea virens	Ascomycota	NCBI
Kluyveromyces lactis	Ascomycota	BROAD-FGI
Lachancea kluyveri	Ascomycota	NCBI
Lachancea thermotolerans	Ascomycota	NCBI
Lachancea waltii	Ascomycota	RFCG
Lodderomyces elongisporus	Ascomycota	BROAD-FGI
Magnaporthe grisea	Ascomycota	BROAD-FGI
Nectria haematococca	Ascomycota	JGI
Neosartorya fischeri	Ascomycota	NCBI
Neurospora crassa	Ascomycota	BROAD-FGI
Paracoccidioides brasiliensis	Ascomycota	NCBI
Penicillium chrysogenum	Ascomycota	NCBI
Penicillium marneffei	Ascomycota	NCBI
Phaeosphaeria nodorum	Ascomycota	NCBI
Pichia pastoris	Ascomycota	NCBI
Pichia guilliermondii	Ascomycota	NCBI
Pneumocystis carinii	Ascomycota	NCBI
Podospora anserina	Ascomycota	NCBI
Pyrenophora tritici-repentis	Ascomycota	BROAD-FGI
Saccharomyces bayanus	Ascomycota	NCBI
Saccharomyces cerevisiae	Ascomycota	NCBI
Saccharomyces kluyveri	Ascomycota	RFCG
Saccharomyces kudriavzevii	Ascomycota	RFCG

Saccharomyces mikatae	Ascomycota	RFCG
Saccharomyces paradoxus	Ascomycota	RFCG
Scheffersomyces stipitis	Ascomycota	NCBI
Schizosaccharomyces cryophilus	Ascomycota	NCBI
Schizosaccharomyces japonicas	Ascomycota	BROAD-FGI
Schizosaccharomyces octoporus	Ascomycota	FGR
Schizosaccharoymces pombe	Ascomycota	BROAD-FGI
Sclerotinia sclerotiorum	Ascomycota	BROAD-FGI
Talaromyces stipitatus	Ascomycota	NCBI
Trichoderma atroviride	Ascomycota	JGI
Trichophyton equinum	Ascomycota	NCBI
Trichophyton rubrum	Ascomycota	NCBI
Trichophyton tonsurans	Ascomycota	NCBI
Trichophyton verrucosum	Ascomycota	NCBI
Uncinocarpus reesii	Ascomycota	BROAD-FGI
Vanderwaltozyma polyspora	Ascomycota	NCBI
Verticillium albo-atrum	Ascomycota	BROAD-FGI
Verticillium dahlia	Ascomycota	BROAD-FGI
Yarrowia lipolytica	Ascomycota	NCBI
Zygosaccharomyces rouxii	Ascomycota	NCBI
Corprinus cinereus	Basidiomycota	BROAD-FGI
Cryptococcus gattii	Basidiomycota	RFCG
Cryptococcus neoformans var. grubiii	Basidiomycota	BROAD-FGI
Cryptococcus neoformans var. neoformans	Basidiomycota	BROAD-FGI
Laccaria bicolor	Basidiomycota	JGI
Malassezia globosa	Basidiomycota	FGR
Malassezia restricta	Basidiomycota	NCBI

Moniliophthora perniciosa	Basidiomycota	NCBI
Phanerochaete chrysosporium	Basidiomycota	JGI
Postia placenta	Basidiomycota	JGI
Puccinia graminis	Basidiomycota	BROAD-FGI
Puccinia triticina	Basidiomycota	NCBI
Schizophyllum commune	Basidiomycota	NCBI
Ustilago maydis	Basidiomycota	BROAD-FGI
Phycomyces blakesleeanus	Zygomycota	NCBI
Rhizopus oryzae	Zygomycota	NCBI
Batrachochytrium dendrobatidis	Chytridiomycota	NCBI
Spizellomyces punctatus	Chytridiomycota	NCBI
Encephalitozoon cuniculi	Microsporidia	NCBI
Encephalitozoon intestinalis	Microsporidia	NCBI
Enterocytozoon bieneusi	Microsporidia	NCBI
Nematocida parisii	Microsporidia	NCBI
Nosema ceranae	Microsporidia	NCBI
Nosema locustae	Microsporidia	NCBI
Octosporea bayeri	Microsporidia	NCBI

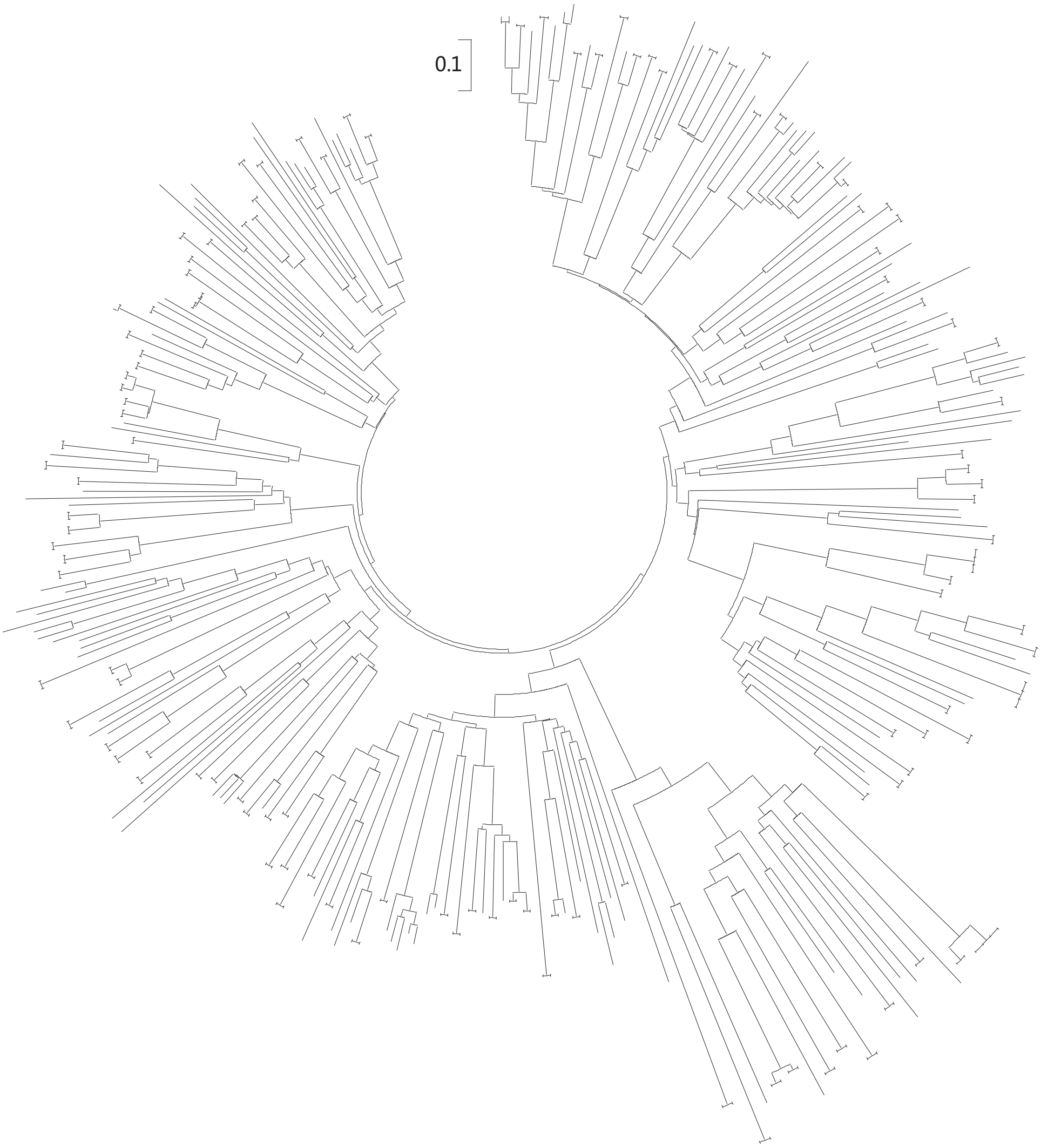

Supplementary figure 1. Neighbor-joining diagram constructed by using all CFEM sequences we identified from 64 fungal species.
